# Statins: a repurposed drug to fight cancer

**DOI:** 10.1186/s13046-021-02041-2

**Published:** 2021-07-24

**Authors:** Wen Jiang, Jin-Wei Hu, Xu-Ran He, Wei-Lin Jin, Xin-Yang He

**Affiliations:** 1 Department of General Surgery, The Affiliated Provincial Hospital of Anhui Medical University, Hefei, 230001 P. R. China; 2 Department of Finance, The First Affiliated Hospital of University of Science and Technology of China (Anhui Provincial Hospital), Hefei, 230001 P. R. China; 3 Institute of Cancer Neuroscience, Medical Frontier Innovation Research Center, The First Hospital of Lanzhou University, The First Clinical Medical College of Lanzhou University, Lanzhou, 730000 P. R. China; 4 Department of General Surgery, The First Affiliated Hospital of University of Science and Technology of China (Anhui Provincial Hospital), Hefei, 230001 P. R. China

**Keywords:** Drug repurposing, Mevalonate pathway, Statins, Synergistic antitumor, Tumor microenvironment

## Abstract

As competitive HMG-CoA reductase (HMGCR) inhibitors, statins not only reduce cholesterol and improve cardiovascular risk, but also exhibit pleiotropic effects that are independent of their lipid-lowering effects. Among them, the anti-cancer properties of statins have attracted much attention and indicated the potential of statins as repurposed drugs for the treatment of cancer. A large number of clinical and epidemiological studies have described the anticancer properties of statins, but the evidence for anticancer effectiveness of statins is inconsistent. It may be that certain molecular subtypes of cancer are more vulnerable to statin therapy than others. Whether statins have clinical anticancer effects is still an active area of research. Statins appear to enhance the efficacy and address the shortcomings associated with conventional cancer treatments, suggesting that statins should be considered in the context of combined therapies for cancer. Here, we present a comprehensive review of the potential of statins in anti-cancer treatments. We discuss the current understanding of the mechanisms underlying the anti-cancer properties of statins and their effects on different malignancies. We also provide recommendations for the design of future well-designed clinical trials of the anti-cancer efficacy of statins.

## Background

Currently, cancer remains the leading cause of death in every country in the world, and the burden of cancer morbidity and mortality is growing rapidly worldwide [1]. The global cancer burden in 2040 is expected to be 28.4 million cases, representing an increase of 47% over 2020 [1]. The development of new drugs to improve cancer treatment can take many years and is extremely expensive [2]. One way to reduce this time-frame and cost is to repurpose existing drugs that show potential cytotoxic activity. Drug repurposing has been used in cancer treatment for many years. Although these drugs have an alternative original indication, they are now being widely used in cancer treatment [3].

Statins are powerful competitive inhibitors of 3-hydroxy-3-methyl-glutaryl-CoA (HMG-CoA) reductase (HMGCR) and are commonly used as lipid-lowering drugs. The source of human plasma cholesterol is either dietary intake or de novo biosynthesis by cells. Statins reduce plasma cholesterol levels by reducing de novo cholesterol biosynthesis and inducing changes in low-density lipoprotein (LDL) receptor expression. However, in the past, studies have shown that statins exhibit pleiotropic effects independent of their lipid-lowering properties. It has been reported that the epigenetic effects of statins mediate these pleiotropic effects, at least to some extent [4]. Among them, the anti-tumor properties of statins have attracted particular attention. For example, Kodach et al. found that statins act as DNA methyltransferase (DNMT) inhibitors, demethylating the bone morphogenetic protein 2 (BMP2) promoter, activating BMP signaling, inducing differentiation of colorectal cancer (CRC) cells, and reducing “stemness” [5]. As repurposed drugs, statins are being investigated for both the prevention and treatment of cancer. Therefore, the anticancer mechanisms of statins have attracted a lot of attention. Among them, the most widely studied is the mevalonate pathway. The flux of the mevalonate pathway is an absolute requirement for all cells, including cancer cells. YAP and TAZ are master transcriptional regulators of normal organ growth and tumor growth [6]. Studies have revealed that the mevalonate pathway promotes YAP/TAZ nuclear localization and activity, while statins impair the YAP/TAZ-dependent transcriptional responses by blocking the mevalonate pathway, thereby inhibiting the development of cancer cells [7]. In addition to the mevalonate pathway, a variety of other anticancer mechanisms of statins have been uncovered one after another.

**Fig. 1:**
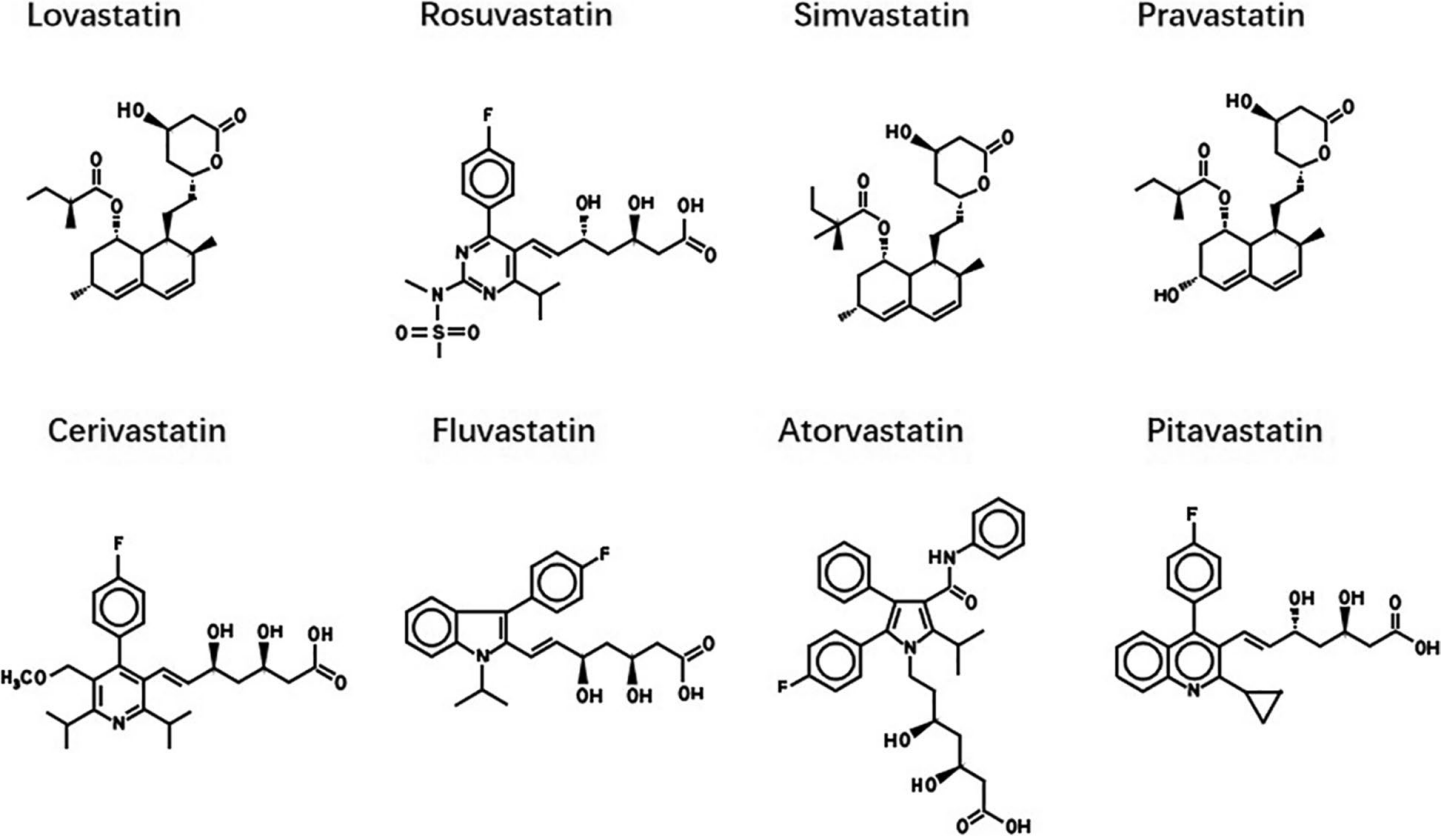
Chemical structures of selected statins

The development of statins as “cholesterol-lowering” drugs began in the mid-1970s. Simvastatin, lovastatin, and pravastatin are all fungal-derived compounds with very similar structures, although the additional hydroxyl groups make pravastatin more hydrophilic than simvastatin or lovastatin. In contrast, fluvastatin, pitavastatin, cerivastatin, atorvastatin and rosuvastatin are synthetic derivative compounds with similarities in structure, such as common fluoride side groups (Fig. 1). Statins may have different intracellular effects depending on their chemical structure [8]. Compared with hydrophilic statins (such as pravastatin and rosuvastatin), lipophilic statins (such as simvastatin) show a greater ability to penetrate the cell membrane and enter hepatocytes and non-hepatocytes through passive diffusion [9, 10]. Furthermore, lipophilic statins have higher pro-apoptotic activity than hydrophilic statins [11]. Due to their higher cytotoxic potential, lipophilic statins may be beneficial in cancer treatment [12].

Here, we review the anti-cancer mechanisms of statins as well as the new perspectives and unique significance of statins in adjuvant cancer therapy to provide a reference for future clinical trials and applications of statins.

## Conventional and off-label of statins

Statins were initially developed to lower lipids and prevent cardiovascular disease. Statins act as competitive inhibitors of HMGCR and can prevent cardiovascular diseases such as atherosclerosis by reducing cholesterol synthesis [13–15]. The effect of statins on cardiovascular morbidity and mortality in patients with and without atherosclerotic disease has been well proven. Furthermore, statins appear to exert pleiotropic effects independent of their lipid-lowering properties, including effects on diabetes, neurological diseases, coronary heart disease, inflammation and cancer [16]. Given the anticancer effects of statins, and the fact that statins are well-tolerated, inexpensive, and less toxic than conventional chemotherapy drugs, many researchers considered repurposing statins as a promising strategy for the treatment of cancer.

## Anti-tumor effects of statins

The anti-cancer properties of statins have attracted considerable interest in recent decades [17]. A growing number of population-based studies and interventional clinical trials indicate that statins produce a series of positive anti-cancer effects (Tables 1 and 2) [56–60].

**Table 1:** Statin use in observational cross-sectional clinical studies

Cancer	Statins	Combination agent	Population	Findings	Ref.
Breast cancers	NR (Before the diagnosis)	NR	7452	The use of statins was associated with increased overall mortality only in patients with ER-positive breast cancer	[18]
	NR (After the diagnosis)	NR	17,880	Statin use after a diagnosis of breast cancer had reduced mortality due to breast cancer and all causes	[19]
	Lipophilic statins and hydrophilic statin	NR	18,769	Simvastatin was associated with a reduced risk of breast cancer recurrence among Danish women diagnosed with stage I–III breast carcinoma	[20]
	Lipophilic statins (After the diagnosis)	NR	1945	The use of lipophilic statins was associated with a reduced risk of breast cancer recurrence	[21]
Prostate cancer	NR (Before the diagnosis)	NR	1022	The use of statins was associated with a decreased risk of prostate cancer	[22]
	Atorvastatin, simvastatin, flovastatin, pravastatin and rosuvastatin (Before the diagnosis)	NR	44,126	The use of statins is associated with lower risk of PTEN-null and lethal prostate cancer	[23]
	Simvastatin, lovastatin, pravastatin, fluvastatin, atorvastatin and rosuvastatin	NR	42,480	The use of statins was associated with a risk reduction overall (6%) and, specifically with advanced PCa (10%)	[24]
	NR	NR	1367	The use of statins was not associated with the risk of advanced prostate cancer	[25]
	NR	NR	1940	The use of statins was associated with the increased risk of prostate cancer	[26]
Pancreatic cancer	Atorvastatin, rosuvastatin, simvastatin, lovastatin and pravastatin	Gemcitabine/paclitaxel	797	The use of statins was associated with better survival in metastatic pancreatic cancer patients who were treated with first-line chemotherapy	[27]
	Atorvastatin, rosuvastatin, simvastatin, lovastatin and pravastatin	NR	2142	The use of statins was associated with lower mortality risk in patients with pancreatic cancer	[28]
Gastric cancer	NR	NR	1835	Statin use may dose-dependently improve the OS of patients with GC after surgery and adjuvant chemotherapy	[29]
	NR	NR	80,271	The use of statins was associated with a reduction of gastric cancer mortality in the general population but not with gastric cancer incidence	[30]
Colorectal cancer	NR (After the diagnosis)	NR	999	The use of statins was associated with reduced risk of death from any cause or from cancer	[31]
	NR	NR	387,518	The use of statins was significantly associated with a decrease in overall mortality and cancer-specific mortality of CRC	[32]
Ovarian cancer	NR (After the diagnosis)	NR	4419	The use of statins was not significantly associated with the reduction of mortality in patients with ovarian cancer	[33]
	Simvastatin, fluvastatin, atorvastatin, pravastatin and rosuvastatin (After the diagnosis	NR	1255	The use of statins was associated with a reduced risk of overall mortality	[34]
Lung cancer	Simvastatin, lovastatin, pravastatin, fluvastatin, atorvastatin and rosuvastatin (After the diagnosis)	EGFR-TKIs	1707	Statins enhance the therapeutic effect and increase survival in patients with lung cancer receiving EGFR-TKI therapy	[35]
	NR (Before and after the diagnosis)	NR	3638	The use of statins (particularly simvastatin) was associated with reduced cancer-specific mortality in patients with lung cancer	[36]
	Atorvastatin, simvastatin, lovastatin, pravastatin, and rosuvastatin (Before and after the diagnosis)	NR	19,974	The use of statins was associated with decreased mortality risk in patients with lung cancer	[37]
Lymphoma	Lovastatin, pravastatin, rosuvastatin, fluvastatin, simvastatin, and atorvastatin (Before the diagnosis)	NR	18,657	The use of statins was associated with a lower risk of NHL	[38]
	NR(Before the chemo-immunotherapy)	NR	4913	The use of statins was associated with the improved survival in newly diagnosed diffuse large B-cell lymphoma	[39]
Glioblastoma	NR	NR	1093	The use of statins was unrelated to OS or PFS of GBM patients	[40]
	NR	NR	2469	The use of statins was not associated with the risk of GBM	[41]
	NR (Before the diagnosis)	NR	280,465	The use of statins was associated with the increased risk of GBM	[42]
Endometrial cancer	NR	NR	77,509	The use of statins was not associated with the risk of endometrial cancer	[43]
Kidney cancer	NR	NR	18,105	Statin use among patients with kidney cancer is associated with significantly improved cancer-specific and overall survival	[44]
Multiple myeloma	NR (Before or after the diagnosis)	NR	4957	The use of statin was associated with a reduced risk of both all-cause and MM-specific mortality	[45]
Head and neck squamous cell carcinoma	NR (After the diagnosis)	NR	1194	Statin use at the time of diagnosis of HPV-negative squamous cell carcinoma (SCC) of the larynx, hypopharynx, and nasopharynx demonstrated improved OS and DSS	[46]

*Abbreviations*: *CRC* Colorectal cancer, *DDS* Disease-specific survival, *EGFR-TKIs* Epidermal growth factor receptor-tyrosine kinase inhibitor, *GBM* Glioblastoma multiforme, *GC* Gastric cancer, *MM* Multiple myeloma, *NHL* Non-Hodgkin lymphoma, *NR* Not reported, *OS* Overall survival, *PCa* Prostate cancer, *PFS* Progression-free survival

**Table 2:** Statin use in interventional clinical anticancer studies

Cancer	Statins	Combination agent	Population	Findings	Ref/CTG.ID
Breast cancer	Fluvastatin (After the diagnosis)	NR	40	Fluvastatin decreased breast tumor proliferation and increased apoptosis	[47]
	Atorvastatin (After the diagnosis)	NR	42	Atorvastatin decreased breast cancer proliferation by influencing the expression of cyclin D1 and p27	[48]
	Simvastatin (After the diagnosis)	FAC	60	Simvastatin may improve the efficacy of FAC in LABC patients	[49]
	Simvastatin (After the diagnosis)	NR	50	NR	NCT03454529
Prostate cancer	Fluvastatin (After the diagnosis)	NR	33	Fluvastatin is associated with promising effects on tumor cell apoptosis	[50]
	Atorvastatin (After the diagnosis)	ADT	400	NR	NCT04026230
Gastric cancer	Simvastatin (After the diagnosis)	Cisplatin/Capecitabine	244	Adding simvastatin to capecitabine–cisplatin did not increase PFS in patients with gastric cancer	[51]
	Pravastatin (After the diagnosis)	ECC	30	Adding pravastatin to ECC did not improve outcome in patients with advanced gastric cancer	[52]
Lung cancer	Simvastatin (After the diagnosis)	Gefitinib	106	Simvastatin may improve the efficacy of gefitinib in that subgroup of gefitinib-resistant NSCLC patients	[53]
	Simvastatin (After the diagnosis)	Irinotecan/Cisplatin	62	NR	NCT00452634
Liver cancer	Pravastatin (After the diagnosis)	Sorafenib	312	Adding pravastatin to sorafenib did not improve survival in patients with advanced HCC	[54]
	Atorvastatin (After the diagnosis)	NR	240	NR	NCT03024684
	Pravastatin (After the diagnosis)	TAE/5-FU	83	Pravastatin prolonged the survival of patients with advanced HCC	[55]
Colorectal cancer	Simvastatin (After the diagnosis)	Cetuximab/Irinotecan	52	NR	NCT01281761
Pancreatic cancer	Simvastatin (After the diagnosis)	Gemcitabine	106	NR	NCT00944463
Ovarian cancer	Simvastatin (After the diagnosis)	NR	20	NR	NCT04457089
Glioblastoma	Atorvastatin (After the diagnosis)	Radiotherapy/Temozolomide	36	NR	NCT02029573
Endometrial cancer	Rosuvastatin (After the diagnosis)	Megestrol acetate	43	NR	NCT04491643
Kidney cancer	Atorvastatin (After the diagnosis)	Zoledronate	11	NR	NCT00490698
Head and neck cancer	Atorvastatin (After the diagnosis)	NR	414	NR	NCT04915183

*Abbreviations*: *ADT* Androgen deprivation therapy, *CRC* Colorectal cancer, *DDS* Disease-specific survival, *ECC* Epirubicin, cisplatin and capecitabine, *EGFR-TKIs* Epidermal growth factor receptor-tyrosine kinase inhibitor, *FAC* Fluorouracil, adriamycin and cyclophosphamide, *GBM* Glioblastoma multiforme, *GC* Gastric cancer, *HCC* Hepatocellular carcinoma, *LABC* Locally advanced breast cancer, *MM* Multiple myeloma, *NHL* Non-Hodgkin lymphoma, *NR* Not reported, *NSCLC* Non-small cell lung cancer, *OS* Overall survival, *PCa* Prostate cancer, *PFS* Progression-free survival, *TAE* Transcatheter arterial embolization, *5-FU* 5-fluorouracil

### Population-based studies

In recent years, a large number of population-based observational studies on the anti-tumor effects of statins have been carried out successively (Table 1); most such studies indicate that statins prolong survival and improve prognosis in cancer patients. A 15-year large-scale observational study of a Danish subgroup showed that the use of statins in cancer patients was associated with a reduction in cancer-related mortality (including 13 cancers) compared with patients who did not use statins [61]. Another retrospective study conducted on 146,326 menopausal women in 40 clinical centers in the USA, with an average follow-up time of 14.6 years, showed that people who currently use statins have a significantly lower risk of cancer death (hazard ratio (HR), 0.78; 95% confidence interval (CI), 0.71–0.86) and all-cause mortality (HR, 0.80; 95% CI, 0.74–0.88) compared with people who have never used statins [57]. A meta-analysis of 1,111,407 cancer patients showed that the use of statins reduced all-cause mortality and cancer-specific mortality by 30% and 40%, respectively [58]. Recently, a study involving 303 patients with advanced pancreatic cancer showed that the use of statins (simvastatin and atorvastatin) was associated with increased overall survival in patients [62]. In addition, in patients receiving radiotherapy, surgery and chemotherapy for advanced pancreatic cancer, statin treatment was associated with a 2-year increase in survival, suggesting that statins help improve the outcome of interventions for advanced pancreatic cancer [63]. A meta-analysis of breast cancer studies showed that the overall use of statins was associated with lower cancer-specific and all-cause mortality. Furthermore, while lipophilic statins were found to be associated with lower breast cancer-specific and all-cause mortality, the protective effect of hydrophilic statins on these outcomes was weak, suggesting that the protective effects of statins on breast cancer are affected by the type of statin [64]. However, in another meta-analysis of 7,858 breast cancer cases, no association between statin use and breast cancer risk was observed at a mean follow-up of approximately 5 years [relative risk (RR), 1.02; 95% CI, 0.89–1.18] [65]. Given that this conclusion is limited by the relatively short follow-up time of the studies analyzed, further studies are required to investigate the effect of long-term statin use on breast cancer risk. In prostate cancer, a study conducted in 4,204 men who underwent prostate biopsy suggested that people who used statins had a significantly reduced risk (8%) of prostate cancer compared with people who did not use statins (RR, 0.92; 95% CI, 0.85–0.98) [22]. Another analysis of 1,001 prostate cancer patients (289 statin users) reported a 0.19 hazard ratio for prostate cancer-specific death among statin users compared with men who did not use statins (95% CI, 0.06–0.56) [66]. A recent clinical study showed that statin use is associated with a reduced risk of phosphatase and tensin homolog (PTEN)-negative and fatal prostate cancer [23]. However, the results of a population-based epidemiologic study showed that the use of statins was not associated with overall prostate cancer risk [67], although the credibility of the results of the study are open to question due to the relatively small sample size and the existence of potential selection and recall biases. In gastric cancer, a recent nationwide cohort study suggested that the use of statins is related to a decrease in gastric cancer mortality in the general population, but has no correlation with the incidence of gastric cancer [30]. A recent prospective observational study of ovarian cancer patients conducted in Australia suggested that the increase in patient survival is related to the use of lipophilic statins, but not to hydrophilic statins [59]. In a large-scale population study of 4,913 patients with diffuse large B-cell lymphoma, statins improved the survival rate in a dose-dependent manner [39]. A study of 999 colon cancer patients showed that the use of statins after diagnosis was significantly associated with a lower risk of death from any cause (adjusted RR, 0.67; 95% CI, 0.51–0.87) and a lower risk of cancer death (adjusted RR, 0.66; 95% CI, 0.49–0.89) [31]. Similarly, a recent meta-analysis showed that statin use was significantly associated with a decrease in overall mortality and cancer-specific mortality of CRC [32]. In liver cancer, a meta-analysis involving 59,073 patients showed that statin use was significantly associated with a reduced risk of hepatocellular carcinoma (HCC) progression compared with those who did not use statins (RR, 0.54; 95% CI: 0.47–0.61) [68].

Inconsistencies in observational studies regarding statin use and cancer morbidity and mortality may be due to significant differences in follow-up periods and potential shortcomings in retrospective and observational studies. For example, statin users might be more health-conscious and might see their doctor more frequently than non-users, so non-users are more likely to be diagnosed with cancer at a later stage compared with statin users. In addition, the race diversity of the population in studies could be limited. Therefore, it is difficult to determine causality from observational studies. Inconsistent findings highlight the importance of well-designed clinical trials to accurately determine the potential of statins as single- or combination-therapy anticancer drugs.

### Interventional clinical trials

In addition to population-based studies, the ability of statins to kill tumors has also been investigated in several interventional clinical trials (Table 2). A perioperative trial showed that high-dose (80 mg/day) neoadjuvant fluvastatin decreased breast tumor proliferation and increased apoptosis compared with low-dose (20 mg/day) treatment [47]. Similarly, another clinical trial in breast cancer patients treated with a high dose of atorvastatin (80 mg/day) in the first 2 weeks before surgery suggested that this treatment decreased breast cancer proliferation by influencing the expression of cyclin D1 and p27 [48]. Recently, Longo et al. revealed that neoadjuvant fluvastatin treatment prior to radical prostatectomy may be effective in inducing intratumoral apoptosis in patients with localized prostate cancer [50].

Statins have also been shown exert antitumor effects by enhancing the response of combination therapy. Recently, Yulian et al. found that simvastatin combined with FAC (Fluorouracil, adriamycin and cyclophosphamide) improved the response to neoadjuvant chemotherapy in patients with advanced local breast cancer [49]. Cantini et al. found that high-intensity statins enhanced the clinical activity of programmed death receptor-1 (PD-1) inhibitors in patients with malignant pleural mesothelioma and advanced non-small cell lung cancer [69]. Han et al. found that simvastatin may improve the efficacy of gefitinib in gefitinib-resistant non-small cell lung cancer (NSCLC) patients [53]. Kawata et al. reported that pravastatin prolonged survival in patients with advanced HCC who received 5-fluorouracil (5-FU) treatment after transcatheter arterial embolization (TAE) [55]. However, Jouve et al. found that adding pravastatin to sorafenib did not improve survival in patients with advanced HCC [54]. Similarly, Kim et al. found that adding simvastatin to capecitabine–cisplatin did not increase PFS in patients with gastric cancer [51]. Konings et al. also found that adding pravastatin to ECC (epirubicin, cisplatin and capecitabine) did not improve outcome in patients with advanced gastric cancer [52].

In summary, the anti-cancer properties of statins have been demonstrated in several clinical and epidemiological studies. However, these effects are not consistent and in several clinical studies, statins were not associated with a reduction in cancer mortality and morbidity, with some even suggesting the opposite effect. Therefore, the anti-cancer properties of statins require further exploration of mechanism of action to maximize design of experiments for further validation in clinical trials.

## Anti-cancer mechanisms of statins

**Fig. 2:**
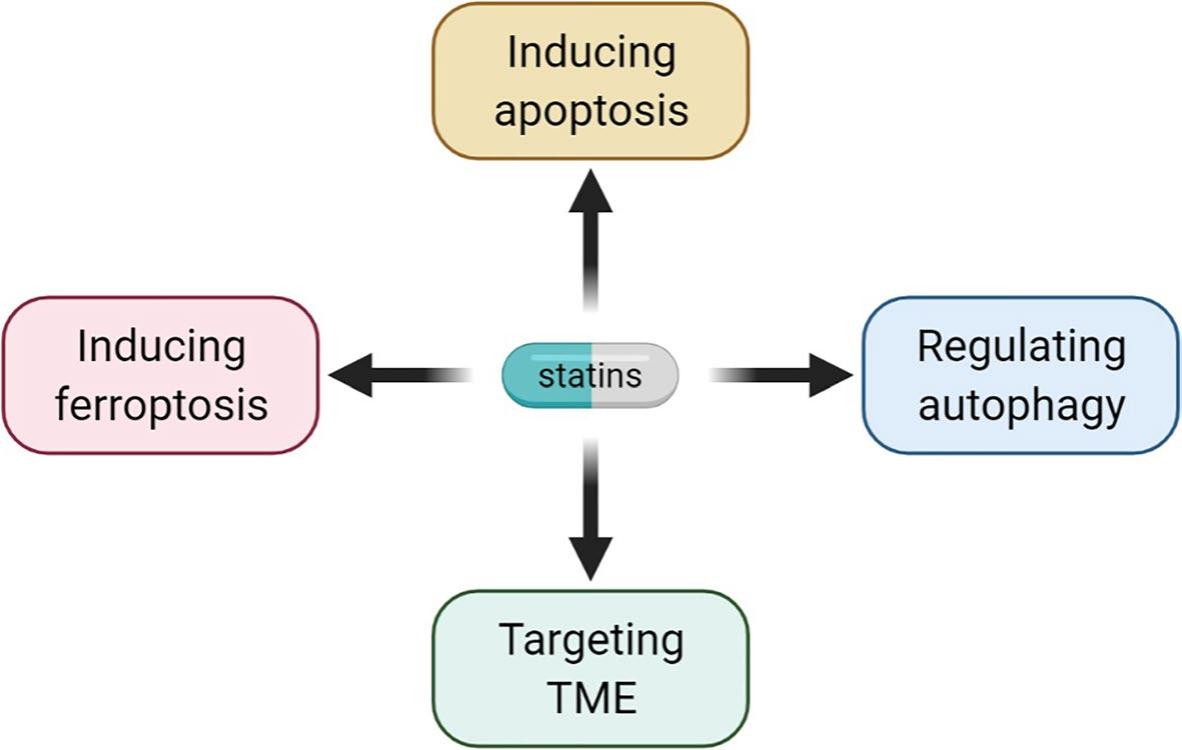
Statin targets in cancer therapy

The anticancer properties of statins have been demonstrated in many preclinical and clinical studies. Therefore, the mechanism underlying the anti-cancer effect of statins has aroused widespread interest. The many anti-cancer mechanisms of statins that have been proposed in recent years are summarized in Fig. 2 and Table 3.

**Table 3:** The anti-cancer effect of statins in preclinical studies

Cancer	Statins	Type of study	Dose of administration	Combination agent	Findings	Ref.
Breast cancers	Simvastatin	In vitro	0–1000 μM	-	Simvastatin induces the apoptosis of MCF-7 cells by increasing caspase-3 and Bax expression	[70]
	Simvastatin	In vitro	0–150 μM	-	Simvastatin induces apoptosis, suppress proliferation and dephosphorylate sequential signaling cascades of PI3K/Akt/mTOR and MAPK/ERK pathways of breast cancer	[71]
	Atorvastatin	In vitro	0–80 μM	-	The anti-proliferative effect of atorvastatin on breast cancer cells is mediated by the induction of both apoptosis and autophagy	[72]
	Simvastatin	In vitro	0–200 μM	-	Simvastatin induces apoptosis via involvement of JNK in breast cancer cells independent of their ER or p53 expression status	[73]
	Lovastatin	In vitro	0–50 μM	-	Lovastatin activates LKB1-AMPK-p38MAPK-p53-survivin cascade to cause MCF-7 cell death	[74]
	Simvastatin	In vitro	0–5 μM	-	Simvastatin increased the expression of miR-140-5p in a dose dependent manner via activating transcription factor NRF1, reduced cell proliferation and induced apoptosis	[75]
	Simvastatin	In vitro	0–100 μM	-	Simvastatin showed the reduction in DNA synthesis and induced cell cycle arrest in the G1 phase in MCF-7 CSCs	[76]
	Mevastatin	In vitro and vivo	0–16 μM (In vitro) 10 mg/Kg (In vivo)	Histone deacetylase inhibitors (HDACi)	Combination treatment inhibited autophagic flux by preventing Vps34/Beclin 1 complex formation and downregulating prenylated Rab7	[77]
	Simvastatin	In vitro	0–100 μM	Doxorubicin	Simvastatin acts synergistically with the anticancer drug doxorubicin against MCF-7 cells through a downregulation of the cell cycle or induction of apoptosis	[78]
	Simvastatin	In vitro	0–100 μM	Pentoxifylline	The combined use of pentoxifylline and simvastatin may drive dormant autophagic cancer cells to undergo apoptosis	[79]
	Simvastatin	In vitro and vivo	0–10 μM (In vitro) 5 mg/kg (In vivo)	-	Simvastatin induces derepression of PTEN expression via NF-κB to inhibit breast cancer cell growth	[80]
	Lovastatin	In vitro	0–100 μM	-	Lovastatin treatment down-regulates the expression of Bcl-2 and activates apoptosis through a mitochondria-operated, ErbB2- regulated mechanism	[81]
Prostate cancer	Simvastatin and fluvastatin	In vitro	0–10 μM	-	Statins decrease cell proliferation and induce cell apoptosis, probably mediated via a downregulation of AKT/FOXO1 phosphorylation in prostate cancer cells	[82]
	Simvastatin	In vitro	0–50 μM	Irinotecan	Combined treatment of simvastatin with irinotecan resulted in enhancement of growth inhibition and induction of prostate cancer cell apoptosis via inhibition of MCL-1	[83]
	Atorvastatin	In vitro	0–50 μM	Irradiation	Atorvastatin enhances the radiosensitivity of hypoxia-induced prostate cancer cells by decreasing the expression of HIF-1α protein	[84]
	Atorvastatin	In vitro	0–5 μM	Caffeine	The combination of atorvastatin and caffeine suppressed proliferation and induced apoptotic death by downregulating phospho-Akt, phospho-Erk1/2, anti-apoptotic Bcl-2 and Survivin protein levels	[85]
	Simvastatin	In vitro and vivo	0–100 μM (In vitro) 2 mg/kg (In vivo)	-	Anticancer efficacy of simvastatin on prostate cancer cells and tumor xenografts is associated with inhibition of Akt and reduced prostate-specific antigen expression	[86]
	Atorvastatin	In vitro and vivo	0–10 μM (In vitro) 5 mg/kg or 10 mg/kg (In vivo)	Celecoxib	The combination of atorvastatin and celecoxib more strongly inhibited growth and the activation of Akt, Erk1/2 and NF-κB in cultured LNCaP cells than either agent alone. In addition, administration of a combination of celecoxib and atorvastatin had a strong inhibitory effect on the progression of androgen-dependent LNCaP prostate tumors to androgen independence in castrated severe combined immunodeficient (SCID) mice	[87]
	Atorvastatin, mevastatin, simvastatin and rosuvastatin	In vitro	0–10 μM	-	Statins reduce the migration and colony formation of PC-3 cells in human bone marrow stroma by inhibiting GGPP production, reducing the formation and the spread of metastatic prostate colonies	[88]
	Atorvastatin	In vitro	0–10 μM	-	Atorvastatin induces autophagy in prostate cancer PC3 cells through activation of LC3 transcription	[89]
	Lovastatin and simvastatin	In vitro	0–2 μM	-	Lovastatin and simvastatin induced apoptosis and cell growth arrest in the G1 phase by inactivating RhoA	[90]
Pancreatic cancer	Pitavastatin	In vitro and vivo	0–0.5 μM (In vitro) 5 mg/kg (In vivo)	Gemcitabine	The combination of gemcitabine and pitavastatin synergically suppressed the proliferation of MIA PaCa-2 cells through causing sub-G1 and S phase cell cycle arrest, activated autophagy and effectively inhibited tumor growth in a nude mouse mode of Mia PaCa-2 xenografts	[91]
Gastric cancer	Simvastatin	In vitro	0–100 μM	-	Simvastatin inhibited the proliferation and migration of NCI-N87 and Hs746T cell lines by reducing mevalonolactone, FPP and GPP	[92]
	Simvastatin	In vitro	0–60 μM	-	Simvastatin inhibits the malignant behaviors of gastric cancer cells by simultaneously suppressing YAP and β-gatenin signaling	[93]
Colorectal cancer	Simvastatin	In vitro	0–20 μM	-	Simvastatin induces the apoptosis of human colon cancer cells and inhibits IGF-1-induced ERK and Akt expression via the downregulation of IGF-1R expression and proapoptotic ERK activation	[94]
	Simvastatin	In vitro and vivo	0–50 μM (In vitro) 20 mg/kg (In vivo)	-	Simvastatin activates the p38MAPK-p53-survivin cascade to cause HCT116 colorectal cancer cell apoptosis	[95]
	Atorvastatin	In vitro and vivo	0–18 μM (In vitro) 15 mg/kg (In vivo)	Nobiletin	Nobiletin/atorvastatin co-treatment synergistically induced extensive cell cycle arrest and apoptosis in colon cancer cells, and the enhanced chemopreventive activities against colon carcinogenesis in rats by the nobiletin/atorvastatin combination were highly synergistic	[96]
Liver cancer	Fluvastatin and pravastatin	In vitro	Fluvastatin: 0–50 μM Pravastatin: 0–500 μM	PBR ligand	Statins inhibited the proliferation of HCC cells by inducing apoptosis and G1/S cell cycle arrest, and the efficacy of treatment with statins was synergistically enhanced by ligands of the peripheral benzodiazepine receptor (PBR)	[97]
	Atorvastatin	In vitro and vivo	0–40 μM (In vitro) 20 mg/kg (In vivo)	-	Atorvastatin induced senescence of hepatocellular carcinoma is mediated by downregulation of hTERT through the suppression of the IL-6/STAT3 pathway	[98]
	Atorvastatin	In vitro	0–30 μM	-	Atorvastatin mediated cell death occurred via inhibition of the PI3K/Akt pathway	[99]
	Simvastatin	In vitro and vivo	0–40 μM (In vitro) 20 mg/kg (In vivo)	-	Simvastatin induced cell cycle arrest through inhibition of STAT3/SKP2 axis and activation of AMPK to promote p27 and p21 accumulation in hepatocellular carcinoma cells	[100]
	Pitavastatin	In vitro	0–20 μM	-	Pitavastatin inhibited growth and colony formation of liver cancer cell, and induced arrest of liver cancer cells at the G1 phase, furthermore, Pitavastatin promoted caspase-9/-3 cleavage in liver cancer cells	[101]
	Simvastatin	In vitro	0–20 μM	Receptor- interacting protein 140	Simvastatin induced the apoptosis of SMCC-7721 cells through the Wnt/β-catenin signaling pathway, as well as that receptor-interacting protein 140 and simvastatin exert a synergistic effect on the inhibition of cell proliferation and survival	[102]
Ovarian cancer	Lovastatin and atorvastatin	In vitro	0–20 μM	-	Statins induce apoptosis in ovarian cancer cells through activation of JNK and enhancement of Bim expression	[103]
	Lovastatin	In vitro and vivo	0–100 μM (In vitro) 12.5 mg/kg (In vivo)	-	Lovastatin affected the expression of genes associated with DNA replication, Rho/PLC signaling, glycolysis, and cholesterol biosynthesis pathways	[104]
Lung cancer	Pitavastatin and fluvastatin	In vitro	0–100 μM	Erlotinib	Cytotoxicity mediated by statin/erlotinib co-treatment is synergistic and can overcome erlotinib resistance in K-ras mutated NSCLC and relies only on apoptosis	[105]
	Simvastatin	In vitro and vivo	0–100 μM (In vitro) 50 mg/kg (In vivo)	-	Simvastatin potently disrupts growth and survival in human SCLC cells by inhibiting Ras signaling	[106]
	Simvastatin	In vitro and vivo	0–1 μM (In vitro) 10 mg/kg (In vivo)	-	Simvastatin prevents proliferation and osteolytic bone metastases of lung adenocarcinoma cells via regulates CD44, P53, MMP family and inactivates of MAPK/ERK signaling pathway	[107]
	Simvastatin	In vitro	0–10 μM	-	Statins break the communication between cancer cells and mesenchymal stromal cells (MSCs) by inhibiting CCL3 secreted by cancer cells and IL-6 and CCL2 produced by MSCs	[108]
Lymphoma	Atorvastatin, fluvastatin and simvastatin	In vitro	0–20 μM	-	Statins induce lymphoma cells apoptosis by increasing intracellular ROS generation and p38 activation and suppressing activation of Akt and Erk pathways, through inhibition of metabolic products of the HMG-CoA reductase reaction including mevalonate, FPP and GGPP	[109]
	Lovastatin	In vitro	0–5 μM	-	Lovastatin inhibits malignant B cell proliferation by reducing membrane cholesterol, intracellular ROS, TRPC6 expression and activity, and intracellular Ca2 +	[110]
Brain cancer	Simvastatin	In vitro	0–20 μM	-	Simvastatin Induces caspase-dependent apoptosis via the mevalonate pathway	[111]
	Simvastatin	In vitro	0–20 μM	Temozolomide	Simvastatin inhibited the autophagy flux induced by temozolomide by blocking autophagolysosome formation	[112]
Melanoma	Simvastatin and atorvastatin	In vitro	0–80 μM	TRAIL	Statins enhanced TRAIL-induced apoptosis, due to suppression of the NF-kB and STAT3-transcriptional targets (including COX-2) and downregulation of cFLIP-L (a caspase-8 inhibitor) protein levels	[113]
	Pitavastatin	In vitro	0–5 μM	Dacarbazine	Combined pitavastatin and dacarbazine treatment activates apoptosis and autophagy resulting in synergistic cytotoxicity in melanoma cells	[114]
	Lovastatin	In vitro	0–4 μM	-	Lovastatin induces apoptosis in multiple melanoma cell lines via a geranylation-specific mechanism through caspase-dependent signaling	[115]
	Simvastatin	In vivo	50 mg/kg	-	Statin lowers PD-1 expression in CD8 + T cells and effectively restores antitumor activity	[116]
Esophageal carcinoma	Simvastatin, lovastatin and pravastatin	In vitro	0–100 μM	-	Statins inhibit proliferation and induce apoptosis in EAC cells via inhibition of Ras farnesylation and inhibition of the ERK and Akt signaling pathways	[117]
Bile duct cancer	Simvastatin	In vitro	0–500 μM	-	Simvastatin suppressed cell proliferation by inducing G1 phase cell cycle arrest in bile duct cancer cells. Furthermore, it induced apoptosis via caspase-3 activation, downregulated the expression of the Bcl-2 protein, and enhanced the expression of the Bax protein. Moreover, simvastatin suppressed the expression of the IGF-1 receptor and IGF-1-induced ERK/Akt activation	[118]
	Simvastatin	In vitro	0–100 μM	-	Simvastatin induces cholangiocarcinoma cancer cell death by disrupting Rac1/lipid raft colocalization and depression of Rac1 activity	[119]
Osteosarcoma	Simvastatin	In vitro and vivo	0–5 μM (In vitro) 40 mg/kg, 50 mg/kg or 80 mg/kg (In vivo)	-	Simvastatin induces apoptosis in osteosarcoma cells via activation of AMPK and p38 MAPK	[120]
	Simvastatin and fluvastatin	In vitro	0–5 μM	-	Statins inhibit GGPP biosynthesis in the mevalonate pathway, and then inhibit signal transduction in the Ras/ERK and Ras/Akt pathways, thereby inhibiting bFGF, HGF, TGF-β expression in LM8 cells	[121]
Head and neck squamous cell carcinoma	Lovastatin	In vitro	0–50 μM	-	Lovastatin activates AMPK-p38MAPK-p63-survivin cascade to cause FaDu hypopharyngeal carcinoma cell death	[122]
	Lovastatin	In vitro	0–10 μM	Chemotherapeutic drugs (cisplatin, cyclophosphamide, doxorubicin and paclitaxel) and Photodynamic therapy	Lovastatin inhibited the CSC properties and induced apoptosis and cell cycle arrest in 5-8F and 6-10B nasopharyngeal carcinoma cell line, and lovastatin conferred enhanced sensitivity to the chemotherapeutic and photodynamic agents in nasopharyngeal carcinoma CSCs	[123]
	Simvastatin	In vitro	0–100 μM	Celecoxib	The combination of celecoxib and simvastatin has showed a significant reduction in tumor cell viability, proliferation and secretion of IL-6 and IL-8	[124]
	Simvastatin	In vivo	5 mg/kg or 10 mg/kg	Monocarboxylate transporter 1 inhibitor	Simvastatin induces metabolic reprogramming in HNSCC mice, reducing lactate production and promoting cancer sensitivity to MCT1 inhibitor	[125]
	Pitavastatin	In vitro	0–0.5 μM	-	Pitavastatin activates the FOXO3a/PUMA apoptotic axis by regulation of nuclear translocation of FOXO3a via Akt/FOXO3a or AMPK/FOXO3a signaling	[126]
Leukemia	Atorvastatin	In vitro	0–80 μM	-	Atorvastatin exerts antileukemia activity via Inhibiting mevalonate-YAP axis in K562 and HL60 cells	[127]
	Simvastatin	In vitro	0–10 μM	-	Simvastatin exhibited anti-leukemic effect in human AML cells in vitro, especially at NRAS^G12D^ mutant AML cell line	[128]
	Simvastatin	In vitro	0–10 μM	Bergamottin	Simvastatin and bergamottin potentially preventing and treating cancer through modulation of NF-kB signaling pathway and its regulated gene products	[129]
Multiple myeloma	Pravastatin	In vitro	0–0.9 μM	-	Pravastatin induces cell cycle arrest in G0/G1 and decreased production of growth factors in Multiple Myeloma cell line	[130]
	Simvastatin	In vitro	0–10 μM	-	Simvastatin induced S-phase cell cycle arrest and apoptosis of multiple myeloma cells through the Chk1–Cdc25A-cyclin A/CDk2 pathway	[131]
Kidney cancer	Simvastatin and fluvastatin	In vitro and vivo	0–2 μM (In vitro) 10 mg/kg (In vivo)	Everolimus	Combination statins and mTOR inhibitor induced a robust activation of retinoblastoma protein via inhibits KRAS or Rac1 protein prenylation, and statins treatment also enhanced the efficacy of an mTOR inhibitor in RCC xenograft models	[132]
Gallbladder cancer	Lovastatin	In vitro and vivo	0–50 μM (In vitro) 50 mg/kg (In vivo)	Cisplatin	Lovastatin sensitized GBC cells to cisplatin-induced apoptosis and suppressed the activation of CHK1, CHK2, and H2AX during DNA damage response, and subcutaneous xenograft mice model suggested lovastatin promoted the therapeutic efficacy of cisplatin, and significantly prolonged the survival times of tumor-bearing mice	[133]
Endometrial cancer	Simvastatin	In vitro	0–12 μM	Metformin	Combination simvastatin and metformin synergistically inhibits endometrial cancer cell viability mediated by apoptosis and mTOR pathway inhibition	[134]

*Abbreviations*: *AML* Acute myeloid leukemia, *CSCs* Cancer stem cells, *EAC* Esophageal adenocarcinoma, *GBC* Gallbladder cancer, *HNSCC* Head and neck squamous cell carcinoma, *MCT1* Monocarboxylate transporter 1, *PBR* Peripheral benzodiazepine receptor, *RCC* Renal cell carcinoma, *TRAIL* TNF-related apoptosis inducing ligand

### The mevalonate pathway

**Fig. 3:**
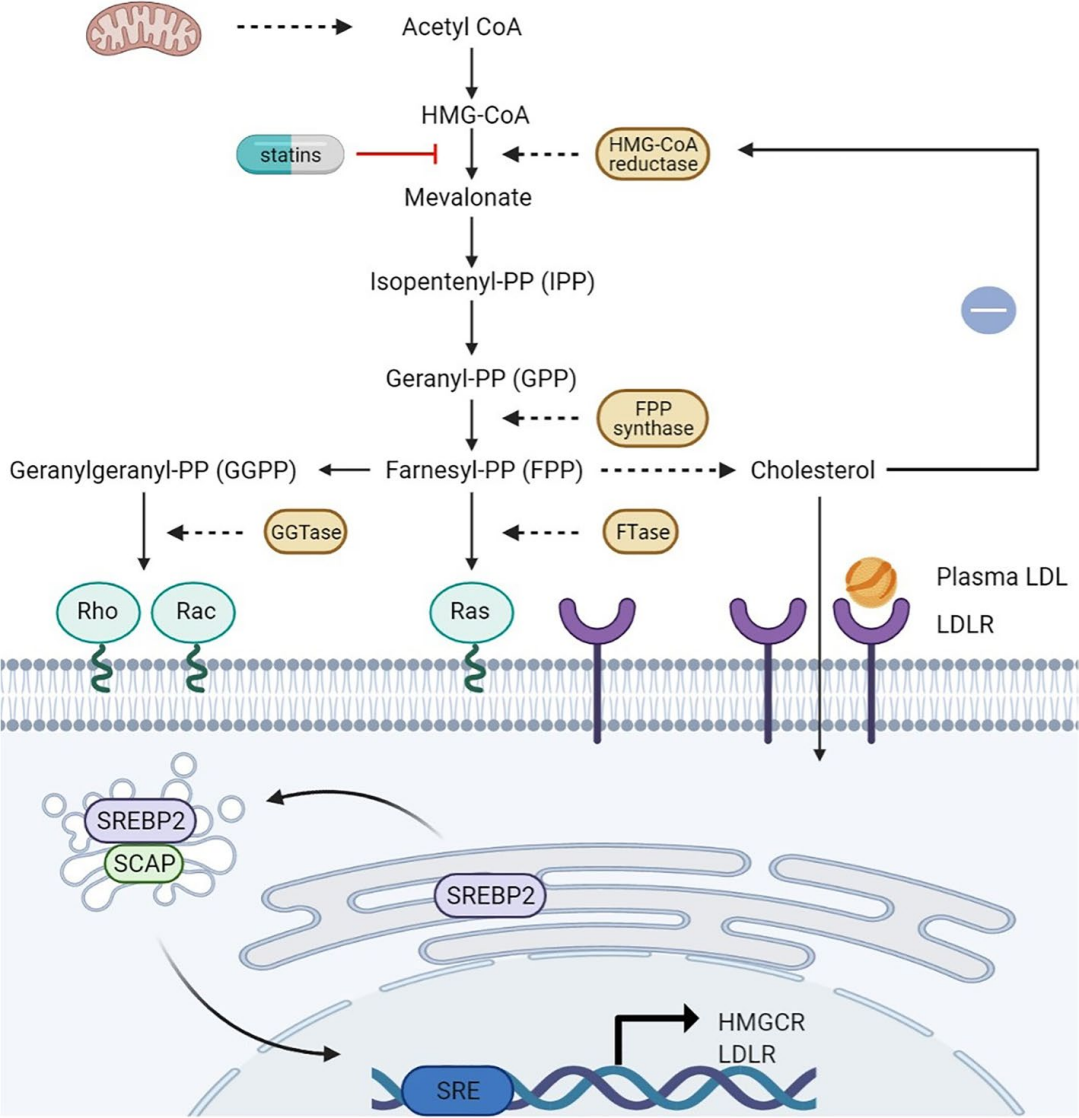
The mevalonate pathway and its transcriptional regulation. The mevalonate pathway begins with the end-product of glycolysis, acetyl-CoA, which is metabolized through several enzymatic steps to mevalonate, IPP, GPP, FPP, GGPP and cholesterol. Both FPP and GGPP can be post-translationally added to proteins, especially small monomeric GTPases (such as Ras, Rho, or Rac). Cholesterol is produced by cells via the mevalonate pathway or LDLR-mediated LDL endocytosis from the serum. When intracellular cholesterol levels are low, SCAP mediates the translocation of SREBP2 to the Golgi apparatus, where it is cleaved by proteases. The active N-terminal fragment is released and translocated to the nucleus, where it binds to the SRE regions of the HMGCR and LDLR promoters to induce gene expression. The inhibitory feedback mechanism mediated by cholesterol is indicated in blue. The mevalonate pathway can be blocked by statins

Metabolic changes are a hallmark of cancer cells [135]. However, it has always been a challenge to identify drugs that target multiple tumor types and cancer-specific metabolic dependence. The mevalonate pathway is an important and complex signaling pathway for the production of various isoprenoids, such as cholesterol, vitamin D, lipoproteins, polyol and ubiquinone [136]. Accumulating evidence indicates that mevalonate pathway (Fig. 3) flux, which is an absolute requirement for all cells, is increased in carcinogenic lesions and is a general feature of cancer [137]. This increased demand for mevalonate is a hallmark of tumorigenesis, and the increased availability of mevalonate pathway intermediates leads to adaptive changes that promote the adaptability of tumor cells [138]. A central motivation for targeting the mevalonate pathway is that selective and well-tolerated inhibitors already exist. Statins block HMGCR synthesis of mevalonate and are prescribed for the treatment of hypercholesterolemia in millions of people. Moreover, numerous in vivo and in vitro studies have shown that statins exert anti-cancer properties by inhibiting the mevalonate pathway. Here, we further divide these effects into those that are induced via non-cholesterol-mediated pathways and cholesterol-mediated pathways.

#### Non-cholesterol-mediated pathways

The mevalonate pathway starts with acetyl-CoA, which is derived from the decarboxylation of pyruvate, the final product of glycolysis. Three molecules of acetyl-CoA are then condensed into 3-hydroxyglutaryl-CoA, which is converted to mevalonate by HMGCR [139] in a reaction that represents the rate-limiting step in this pathway. Mevalonate is then phosphorylated by mevalonate kinase and metabolized to isopentenyl pyrophosphate (IPP) [140–142], which is the factor that defines the key role of the mevalonate pathway in the synthesis of farnesyl pyrophosphate (FPP) and geranylgeranyl pyrophosphate (GGPP) via reactions that are catalyzed by farnesyl diphosphate synthase (FDPS) and GGPP synthase (GGPPS) [141, 143]. FPP is the basic component required for the production of squalene, which is a precursor of cholesterol and a key product of the mevalonate pathway. Cholesterol is mediated by additional enzymes, such as squalene epoxidase and squalene synthase [144, 145]. Cholesterol plays a vital role in the establishment and maintenance of the structure and function of cellular membranes. It is also a precursor of steroid hormones, vitamin D and bile acids [146, 147]. In addition, cholesterol is essential for the biogenesis of lipid rafts composed of lipids and proteins. These dynamic assemblies float within the cell membrane and participate in membrane transport, signal transduction, and cell polarization [148]. The synthesis of FPP and GGPP is essential for the post-translational modification referred to as prenylation of various proteins. This reaction is mediated by farnesyltransferase (FTase) I and geranyltransferases (GGTases) I and II. The localization, membrane anchoring and function of hundreds of signaling proteins depend on post-translational prenylation [139, 149]. These proteins include small monomeric GTPases (guanosine-triphosphate hydrolase) (e.g. Ras, Rho or Rac) and the γ-subunit of G-protein coupled receptors, which are involved in numerous important intracellular signaling pathways [150]. These small monomeric GTPases promote their anchoring and activation on the plasma membrane through isoprenylation and association of these signaling molecules with effectors to regulate a wide range of cellular functions, such as endocytosis/exocytosis, differentiation, cytoskeletal rearrangement and contraction, migration, apoptosis, and proliferation [151, 152]. Inhibition of HMGCR by statins depletes the pools of mevalonate (MA), IPP, FPP and GGPP in cells. This process interferes with the function of Ras and Rho family GTPases [153–157]. Indeed, lower GGPP and FPP concentrations, leading to reduced RAS and Rho isoprenylation, signal transduction, and DNA synthesis, are important functional consequences of statins in the treatment of cancer [119, 158–162]. Inhibiting the farnesylation of these proteins restricts their activity and might hinder cancer cell proliferation [163, 164]. Studies have shown that the inhibitory effect of simvastatin on the proliferation, migration and invasion of the gastric cancer cell lines MKN45 and MGC803 is due to a reduction in GGPP and RhoA activity mediated by inhibition of β-catenin and the Yes-associated protein (YAP) signaling pathways. Statins activate the intrinsic apoptotic pathway by disrupting the prenylation of key regulatory proteins. Therefore, the depletion of isoprenoids leads to the apoptosis of a variety of cancer cells [112, 165]. Simvastatin has also been shown to significantly inhibit the proliferation and migration of two cell lines of intestinal (NCI-N87) and diffuse (Hs746T) metastatic gastric tumor histological subtypes. Furthermore, the anti-proliferative effects of simvastatin in gastric cancer cells were reversed by the addition of mevalonate, FPP and GPP [92]. Simvastatin also activates caspase-3/-7/-9, which in turn induce apoptosis in human cancer cell lines through depletion of isoprenoids, a precursor to the prenylation of the small Rho GTP-enzyme [73, 166]. Fujiwara et al. reported that statins promote cell death by increasing the activation of caspases-3/-9, inducing Bcl-2 interacting mediator of cell death (Bim) expression, arresting the cell cycle at the G1 phase, and by decreasing the mitochondrial membrane potential (Δψm) through inhibition of Ras/ERK and Ras/mTOR pathways [167]. Statins stimulate membrane FasL expression and lymphocyte apoptosis via the RhoA/Rho-associated protein kinase (ROCK) pathway in murine melanoma cells in vitro [168]. A recent study showed that statins inhibit pyrimidine nucleotide biosynthesis by inhibiting the mevalonate pathway, and also induce oxidative stress and apoptosis in p53-deficient cancer cells [169]. Statin-induced GGPP depletion blocks macropinocytosis and starves cells with oncogenic defects [170]. Lovastatin-induced MCF-7 cancer cell death is mediated via the liver kinase B1 (LKB1)-AMP-activated protein kinase (AMPK)-p38 mitogen-activated protein kinase (p38MAPK)-p53-survivin signaling cascade [74]. Yin et al. found that pituitary tumor-transforming gene 1 (PTTG1) was significantly overexpressed in malignant breast cancer cell lines, and simvastatin downregulates its expression by inhibiting GGPP [171]. Freed-Pastor et al. revealed that mutant p53 can disrupt mammary acinar morphology by up-regulating the mevalonate pathway, thereby exerting a carcinogenic effect, while statins inhibit the mevalonate pathway to exert a tumor suppressor effect [172]. Epithelial-to-mesenchymal transition (EMT), which is an effective mechanism of cancer metastasis, is a dynamic multi-gene programming cycle [173]. Lipophilic statins have been found to function as antagonists of EMT signaling pathways in stem-like cells in breast cancer by inhibiting the mevalonate pathway [174]. In prostate cancer, lovastatin and simvastatin inactivate RhoA, thereby inducing cell apoptosis and cell cycle arrest in the G1 phase [90].In ovarian cancer, statins promote cell apoptosis in a dose-dependent manner via a GGPP-mediated mechanism [175]. Accumulating evidence shows that statins inhibit tumor metastasis by disrupting geranylgeranylation and farnesylation of small GTPases [160, 176–178]. These findings support the view that statins may be promising anti-cancer drugs.

#### Cholesterol-mediated pathways

A link between cellular cholesterol levels and cancer was first reported a century ago [179]. Since then, several studies have shown that cholesterol levels in tumors are elevated compared to those in normal tissues [180, 181].

Variation in the mechanisms by which tumor cells increase intracellular cholesterol have been observed, including increased expression of low-density lipoprotein receptor (LDLR) or insufficient feedback regulation of LDL [182–188]. Cholesterol promotes the progression of sex hormone-responsive breast cancer and prostate cancer by providing estrogen and androgens [189]. In addition, cholesterol is very important for cell proliferation and the cell cycle, especially for progression to S-phase [190], and plays a central role in lipid rafts. Cancer cells have an increased requirement for cholesterol and contain more lipid rafts than normal cells to meet the need for tumor-promoting cell signaling proteins [147]. Cells acquire cholesterol from plasma via LDLR-mediated endocytosis, or synthesize cholesterol de novo via the mevalonate pathway. The discovery of sterol regulatory element binding proteins (SREBPs) is a breakthrough in understanding the regulation of mevalonate pathway genes. These transcription factors are synthesized as inactive precursors on the endoplasmic reticulum membrane, where they bind to SREBP cleavage activating proteins (SCAPs), which function as sterol sensors. SREBP-2 is transported to the Golgi apparatus by SCAPs when intramembranous cholesterol levels are reduced. SREBPs are cleaved by two proteases in the Golgi apparatus, and then their active fragments are released and translocated to the nucleus, where they bind to the sterol regulatory elements (SRE) in the promoter regions of the *HMGCR* and *LDLR* genes to induce their expression. Transcriptional activation of HMGCR leads to the de novo biosynthesis of cholesterol via the mevalonate pathway. After *LDLR* transcription is activated, receptor-mediated endocytosis of low-density lipoprotein leads to increased cellular cholesterol uptake. When the intracellular cholesterol level is high, SCAPs prevent the translocation and activation of SREBPs, resulting in the transcriptional inactivation of *HMGCR* and *LDLR* [191–194]. The complexity of these precisely controlled regulatory mechanisms ensures that cholesterol homeostasis is maintained within cells [195]. Dysregulation of cholesterol homeostasis or key molecules in cholesterol are not only related to several well-known carcinogenic pathways, but also related to inflammasome- and miRNA-mediated cancer development. SREBP2 not only regulates the transcriptional activity of cholesterol biosynthesis genes and LDLR-mediated cholesterol influx, but also regulates the transcriptional activity of Nod-like receptor protein 3 (NLRP3) inflammasome-related inflammation. The causal relationship between chronic inflammation and cancer is widely established. Inflammasomes, which are large intracellular multi-protein signaling complexes formed in response to inflammation, participate in the activation of inflammatory protease caspase-1 and the pro-inflammatory cytokines interleukin (IL)-1β and IL-18 [196]. NLRP3 regulation is closely related to the development and progression of tumors, including head and neck squamous cell carcinoma [197], colorectal cancer [198] and breast cancer [199]. MicroRNA 33 (miRNA33) binds to the *SREBP2* gene to positively regulate SREBP2 expression. Hyperactivation of cholesterol biosynthesis leads to uncontrolled cell growth [200].

Newly synthesized free cholesterol is transported to subcellular membranes by cholesterol transfer protein; however, to avoid excessive accumulation of free cholesterol, surplus cholesterol is esterified by acyl-CoA:cholesteryl acyltransferase 1 (ACAT1) to form cholesteryl esters (CEs), which are stored as lipid droplets (LDs) in cells [201]. Although CEs serve as a cholesterol reservoir, the accumulation of CEs or ACAT1 overexpression promote tumorigenesis. In a xenograft model of glioblastoma, ACAT1 ablation was shown to reduce tumor progression [202]. ACAT1 overexpression was also found in hepatocellular carcinoma [203]. In addition, the migration ability of breast cancer cells and the progression of prostate cancer was found to be suppressed by inhibiting the expression of ACAT1 [204, 205]. When de novo biosynthesis is the main source of intracellular cholesterol, most cells acquire cholesterol from LDL in the circulatory system via LDLR-mediated endocytosis [206]. Proprotein-convertase-subtilisin-kexin type-9 (PCSK9) overexpression promotes lysosomal degradation of LDLR [207], leading to hypercholesterolemia and ultimately, the occurrence of hepatocellular carcinoma [208]. Excess cholesterol also produces oxysterols, which are natural ligands for liver X receptors (LXRs). The binding of cholesterol to LXRs triggers conformational changes of the receptor, thereby enhancing the interaction with the co-activator protein and promoting the transcription of cholesterol efflux-related genes [209], including ATP-binding cassette (ABC) subfamily A member 1 (ABCA1), ABC subfamily G member 1 (ABCG1) and ABCG5/8. The excess cholesterol can be exported to lipid-poor apolipoprotein A-I (ApoA-I) via ABCA1 or ABCG1, and generate high-density lipids proteins (HDLs), which are transported back to the liver [210–213]. When the intracellular cholesterol level is high, the LXR can upregulate *ABCA1* transcription [213]. However, in cancer cells, ABCA1 expression is inhibited via the phosphatidylinositol-3-kinase (PI3K)/protein kinase B (AKT)/mechanistic target of rapamycin complex 1 (mTORC1) pathway [200]. MiR-183 promotes the proliferation and anti-apoptotic properties of colon cancer cells by directly degrading ABCA1 mRNA to maintain high levels of intracellular cholesterol [214]. Similarly, miR-27a-3p also inhibits cancer cell apoptosis by blocking cholesterol efflux or targeting ABCA1 [215]. LXR overexpression has been shown to have anti-proliferative effects in gastric cancer cells [216]. Cholesterol and its oxygenated derivatives bind with high affinity to the G protein-coupled receptor (GPCR) known as smoothened receptor (SMO), which activates the Sonic Hedgehog (SHH) pathway [217]. The SHH pathway is considered to be an oncogenic signaling cascade based on its ability to promote cell cycle progression and stem cell proliferation by increasing glioma-associated oncogene homolog 1 (GLI1) activity, which subsequently activates hedgehog targeting genes, thereby enhancing tumor formation [218]. Statins arrest SHH signaling in medulloblastoma cells and fibroblasts by inhibiting cholesterol synthesis, thus attenuating tumor proliferation [219]. Similarly, Fan et al. recently found that statins suppress medulloblastoma growth without bone toxicity by repressing hedgehog signaling in tumor cells [220]. These studies illustrate the inseparable correlation between cholesterol and cancer, and reveal that statins exert anti-cancer properties by inhibiting the de novo synthesis of cholesterol.

### Statins regulate autophagy

Both autophagy and apoptosis are important biological phenomena involved in various processes such as development and growth. The major distinction between autophagy and apoptosis is their purpose. For example, during stress conditions, autophagy primarily targets certain toxic components to preserve cell survival [221, 222]. When autophagy is insufficient to reduce stress and compensate for cellular damage, this results in autophagic cell death, which is similar to apoptosis [223, 224]. Since the discovery of autophagy, numerous studies have shown that autophagy is associated with the progression of pathological conditions, especially cancer [225–227]. Autophagy has been shown to reduce the viability of tumor cells or play a protective role in cancer. Thus, autophagy seems to play a dual role in tumorigenesis and tumor progression [228]. The tumor-suppressive function of autophagy is primarily important in the early stages of cancer development, representing a critical quality control mechanism that maintains genomic integrity by regulating the degradation of damaged proteins or organelles (such as dysfunctional mitochondria) [228, 229]. Autophagy also facilitates oncogene-induced senescence [230] and contributes to cancer immune surveillance [231]. The tumor-promoting effects of autophagy are exerted mainly in the later stages of tumor development. Due to the rapid proliferation of cancer cells, the nutritional requirements for anabolic pathways are high. Autophagy contributes to cancer cell metabolism by recycling cellular substrates [232]. Autophagy also promotes the survival of cancer cells under stress conditions, such as tumor hypoxia, nutrient deprivation or endoplasmic reticulum (ER) stress [233, 234]. In summary, interfering with autophagy is a reasonable therapeutic strategy to improving the effect of anti-cancer treatments in the clinic.

A variety of anti-cancer drugs have been developed based on their ability to regulate autophagy. The first report of statin-induced autophagy showed that hydrophobic statins, such as simvastatin, induced autophagy in rhabdomyosarcoma A204 cells [235] through depletion of the GGPP pool [236]. After deletion of the autophagy-related gene 5 (ATG5) or ATG7 gene in SPC-A-1 cells, fluvastatin inhibits bone metastasis by inducing autophagy in lung adenocarcinoma cells [237]. In HEK293 human embryonic kidney cells, atorvastatin induces autophagy in a mevalonate (MEV) pathway-dependent manner by inhibiting GGPP synthesis [89]. Autophagy was induced in U251 cells by treatment with simvastatin for 24 h, and this effect was reversed by the addition of MEV, which again suggests that the MEV cascade is involved in statin-mediated autophagy [238]. In human leukemia cells treated with simvastatin for 24–72 h, the induction of autophagy flux appeared to be mediated by the inhibition of cholesterol production, rather than the synthesis of FPP or GGPP [239]. Atorvastatin induced autophagy in the Huh7 and HCT116 gastrointestinal tumor cell lines independent of the MEV pathway [240]. Simvastatin can impair autophagy flux and induce breast cancer cell death by stimulating the extracellular regulated protein kinases 1 and 2 (ERK1/2) and Akt pathways [79]. Lovastatin stimulates autophagy through the Rac/phospholipase C/inositol 1,4,5-triphosphate axis, and markedly decreases the viability and migration ability of malignant pleural mesothelioma tumor cells [241]. Due to the resistance of tumor cells to apoptosis-based therapy, and the stimulatory effect of cancer cell apoptosis on the survival and proliferation of neighboring cells, the induction of autophagic cell death is preferred to apoptosis as a strategy to combat cancer. Studies have shown that the combination lovastatin and farnesyltransferase inhibitors can stimulate non-apoptotic cell death and impair autophagy flux [242]. Angiogenesis plays a critical role in cancer progression. Atorvastatin stimulates autophagy in human umbilical vein endothelial cells. Furthermore, high-dose atorvastatin activates apoptosis and inhibits angiogenesis. Atorvastatin stimulates autophagy through upregulation of light chain 3-phosphatidylethanolamine conjugate (LC3II), which effectively reduces proliferation and viability of cancer cells [243]. The benefits of statins as adjuvant therapy for cancer have been widely reported. The combination of lovastatin and cisplatin has been found to enhance expression of the autophagy marker LC3B-II and decrease the viability of cancer cells through the induction of autophagic cell death [244]. Autophagy has been shown to play a central role in the induction of chemotherapeutic resistance [245, 246]. The anti-cancer effects of the chemotherapeutic agent temozolomide (TMZ), which is used to treat glioblastoma (GBM), are markedly reduced by TMZ-induced autophagy. A recent study showed that simvastatin inhibits the autophagic flux induced by TMZ by blocking autophagolysosome formation, thereby sensitizing glioblastoma cells to TMZ-induced cell death [112]. However, a recent study showed that atorvastatin reduces cell viability and promotes cervical cancer cell apoptosis by inducing the activation of caspase-3 and poly (ADP-ribose) polymerase (PARP) and upregulating Bim. However, atorvastatin induced cellular autophagy in cervical cancer cells, and pharmacologic inhibition of autophagy using autophagy inhibitors remarkably enhanced atorvastatin-induced apoptosis of cervical cancer cells [247].

In summary, statins are capable of modulating autophagy, making them promising candidates for the treatment of cancer. However, the regulatory effects of statins on autophagy require further clarification.

### Statins induce ferroptosis

The term ferroptosis, which was coined in 2012 [248], is a form of programmed cell death (PCD) that differs from apoptosis, necrosis, pyroptosis and autophagy [249, 250]. Ferroptosis is associated with the occurrence of multiple diseases [251, 252] and its role in the treatment of cancer has attracted increasing attention [253–258]. In essence, ferroptosis is a process that occurs as a result of metabolic dysregulation, and is characterized by iron overload, lipid reactive oxygen species (ROS) accumulation, and lipid peroxidation [248]. Ferroptosis is inhibited by sequestration of free iron, inhibition of polyunsaturated fatty acid (PUFA) synthesis, or scavenging of ROS. Ferroptosis and lipid peroxidation are mainly controlled by three parallel systems: the glutathione (GSH)/glutathione peroxidase 4 (GPX4) axis, the ferroptosis suppressor protein 1 (FSP1)/ubiquinone (CoQ10)/NAD(P)H axis and the GTP cyclohydrolase 1 (GCH1)/tetrahydrobiopterin (BH4)/phospholipid axis [259–261]. The induction of ferroptosis in tumor cells is a promising anti-tumor strategy.

**Fig. 4:**
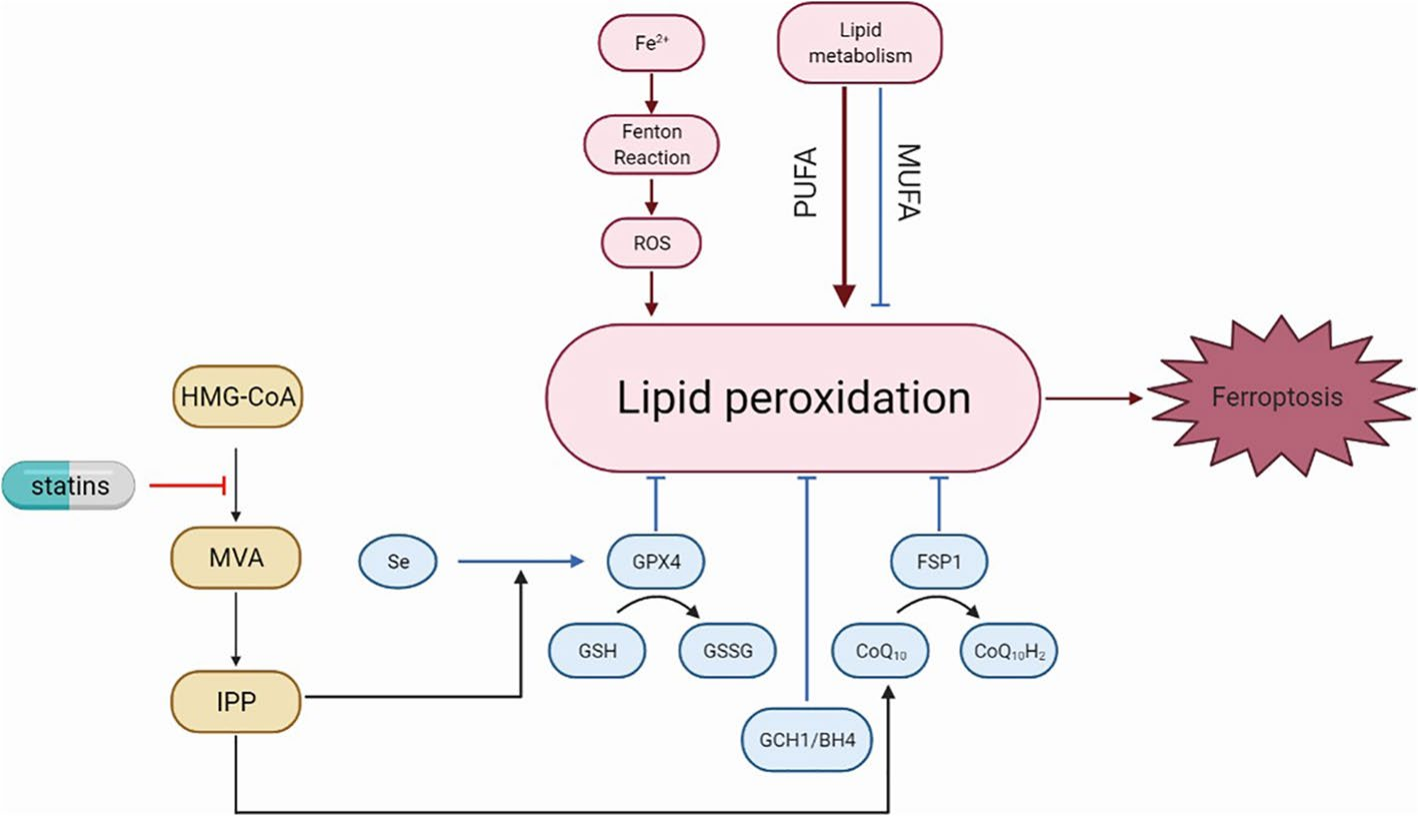
The relationship between statins and ferroptosis. Iron overload, lipid reactive oxygen species (ROS) accumulation, and lipid peroxidation are prerequisites for the activation of cell death by ferroptosis. Ferroptosis is governed by three antioxidant axes, i.e., the GSH/GPX4, FSP1/CoQ10/NAD(P)H and GCH1/BH4 axes. The FSP1/CoQ10/NAD(P)H axis relies on the mevalonate pathway to generate CoQ10. IPP, the precursor of CoQ10, is also a limiting substrate for enzymatic isopentenylation of Sec-tRNA, thereby influencing the expression of GPX4. Statins regulate the GSH/GPX4 and FSP1/CoQ10/NAD(P)H axes via the mevalonate pathway, thereby inducing cell death by ferroptosis

Statins are associated with ferroptosis via the mevalonate pathway (Fig. 4), which is closely related to the regulation of the GSH/GPX4 and FSP1/CoQ10/NAD(P)H axes. The mevalonate pathway is crucial for the synthesis of GPX4 itself and generation of the CoQ10 backbone. IPP produced by the mevalonate pathway is the precursor of CoQ10.IPP positively regulates Sec-tRNA, which functions as a key regulatory element during the maturation of GPX4 [262, 263]. Blocking the rate-limiting enzyme in the mevalonate pathway by using statins compromises the efficient translation of GPX4 and consequently sensitizes cells to ferroptosis [264, 265]. It was recently revealed that the mechanism underlying the protective effect of CoQ10 is based on the ability of FSP1 to use CoQ10 as a substrate to hinder lipid autoxidation [259, 260]. A recent study showed that drug-resistant cancer cells in a high-mesenchymal cell state were sensitive to ferroptosis induced by GPX4 inhibition or statin treatment. Fluvastatin treatment decreased GPX4 expression in a time- and concentration-dependent manner and its effects are enhanced by combination with the direct GPX4 inhibitor RSL3 [264]. Therefore, in the absence of bioavailable GPX4 inhibitors, statins stand out as candidates for the therapeutic induction of ferroptosis in highly mesenchymal and chemotherapy-resistant cancer cells.

### Statins induce pyroptosis

Pyroptosis is an inflammatory form of PCD that is distinct from autophagy and ferroptosis. It was first described in myeloid cells infected by pathogens or bacteria in 1992 [266–268]. Pyroptosis is characterized by cell swelling, rupture, lysis and release of pro-inflammatory molecules, such as IL-1β and IL-18 [269, 270] and is induced by members of the gasdermin superfamily, including GSDMA, GSDMB, GSDMC, GSDMD, and GSDME [271–276]. The inflammasome activates caspase family proteins to cleave gasdermin. The resulting active form of the protein translocates to the cell membrane, forming pores that lead to cell swelling, cytoplasmic outflow, cell membrane rupture and eventually cell pyroptosis [273–275]. Generally, GSDMD, which is downstream of inflammasome activation, is cleaved by inflammatory caspases (caspase1/4/5/11) to induce pyroptosis, while GSDME is cleaved by apoptotic caspases (caspase 3) to induce pyroptosis [272]. There are two main pathways of pyroptosis: the caspase-1-mediated canonical pathway and the caspase-4/5/11-mediated non-canonical pathway. In the canonical pathway, danger is sensed through inflammasomes, leading to the recruitment and activation of caspase-1, which activates inflammatory factors such as IL-18 and IL-1β and cleaves the N-terminal sequence of GSDMD. The activated form of GSDMD then bind to the cell membrane to generate pores and induce pyroptosis [277]. In the non-canonical pathway, human homologs caspase-4, 5 and murine caspase-11 recognize and bind to the bacterial lipopolysaccharide (LPS) and then cleave GSDMD, leading indirectly to caspase-1 activation and pyroptosis [271, 274].

Many studies have shown that pyroptosis is closely related to the occurrence and development of various diseases, such as cancer [267, 278, 279]. For example, recent studies have shown that the low GSDMD expression significantly promotes the proliferation of gastric cancer cell both in vivo and in vitro [280]. Zhou et al. demonstrated that that iron supplementation at appropriate doses in iron-deficient patients is sufficient to maximize the anti-tumor effects of clinical ROS-inducing drugs, and inhibit the growth and metastasis of melanoma cells through GSDME-dependent pyroptosis [281]. A recent study showed that atorvastatin inhibits pyroptosis through the long non-coding RNA (lncRNA) NEXN-AS1/NEXN pathway in human vascular endothelial cells [282]. In a mouse model of cardiac injury induced by coronary microembolization, rosuvastatin decreased the expression of NLRP3, caspase-1, interleukin-1β and GSDMD N-terminal domains, suggesting that this drug can protect against this type of cardiac injury by inhibiting pyroptosis [283]. However, the potential role of pyroptosis in the anti-tumor effects of statins remains to be fully elucidated.

### Statins target the TME

**Fig. 5:**
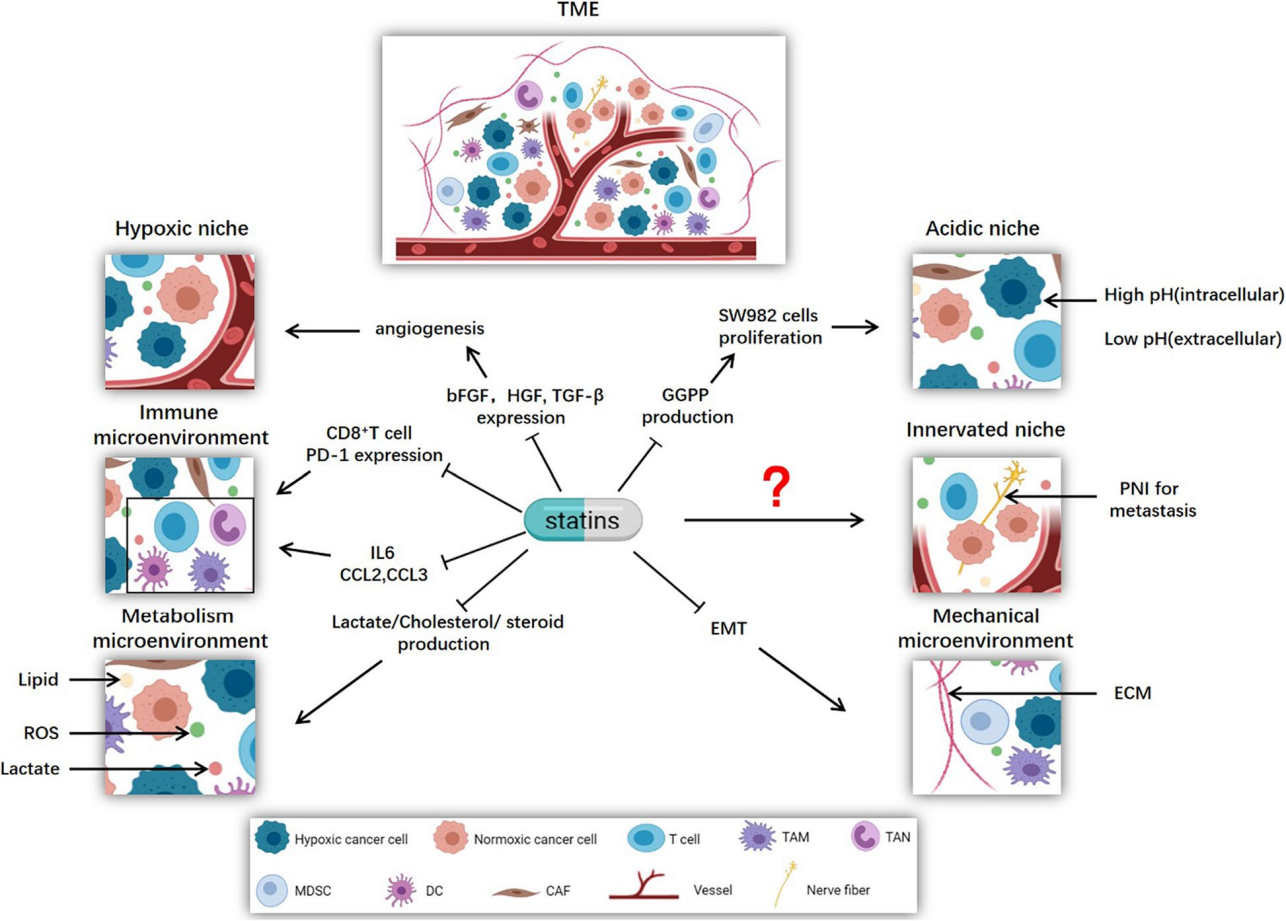
Statins fight cancer by targeting TME. TME can be classified into six specialized microenvironments: hypoxic niche, immune microenvironment, metabolism microenvironment, acidic niche, innervated niche, and mechanical microenvironment. Statins exert anticancer properties by targeting these specialized microenvironments

Tumor microenvironment (TME) denotes the non-cancerous cells and components presented in the tumor, including molecules produced and released by them (Fig. 5). Over time, cancer has been recognized as an evolutionary and ecological process that involves constant, dynamic, and reciprocal interactions between cancer cells and TME [284]. The constant interaction between tumor cells and the TME plays a decisive role in tumor initiation, progression, metastasis, and response to therapy. The acquisition and maintenance of the hallmarks of cancer, such as maintained proliferation signaling, resistance to cell death, angiogenesis, activation of invasion and metastasis, and the induction of tumor-promoting inflammation, as well as the evasion of immune destruction, depend to varying degrees on the contribution of TME.

Given our increased understanding of the crucial roles of the TME in tumor development and therapeutic resistance, the focus of cancer research and treatment has gradually switched from a cancer-centric model to a TME-centric model. Efforts have been made to target components of the TME to achieve therapeutic benefits for cancer patients. We have introduced some specialized microenvironments in TME, focusing on hypoxic niche, immune microenvironment, metabolism microenvironment, acidic niche, innervated niche, and mechanical microenvironment (Fig. 5) [285]. Compared with the whole TME, a specialized microenvironment seems to be a better target for cancer treatment. The anti-tumor effects of some conventional drugs have been shown to be mediated by targeting the TME. Statins have been observed to exert anti-tumor effects by targeting these specialized microenvironments.

In the metabolic microenvironment, simvastatin has been shown to induce metabolic reprogramming in a mouse model of head and neck squamous cell carcinoma (HNSCC) by reducing the production of lactic acid and cancer sensitivity to monocarboxylate transporter 1 (MCT1) inhibitor, thereby inhibiting HNSCC [125].

In the mechanical microenvironment, simvastatin repolarizes tumor-associated macrophages (TAM) and promotes M2-to-M1 phenotype switching of macrophages via cholesterol-associated LXR/ABCA1 regulation, thereby remodeling the tumor microenvironment and inhibiting EMT [286].

Statins are also reported to target the immune microenvironment through cytokines or chemokines and immune checkpoints. Statins inhibit the survival of lung cancer cells by inhibiting the secretion of CCL3 by lung cancer cells as well as IL-6 and CCL2 secretion by mesenchymal stromal cells, indicating the potential of statins as repurposed drugs for targeting the immune TME [108]. In inflammatory breast cancer (IBC), simvastatin blocks the activation of mesenchymal stem cells by decreasing IL-6 production [287]. A recent study showed that simvastatin can enhance the anti-tumor activity of CD8^+^ T cells by reducing cholesterol in the TME [116].

Hypoxia is induced in tumors by the outgrowth of cancer cells and unmatched angiogenesis and oxygen supply, accompanied by changes in the rate of cancer cell metabolism. Hypoxia activates vascular endothelial cells, upregulates vascular endothelial growth factor (VEGF) transcription, and stimulates excessive angiogenesis, thereby influencing the TME and therapeutic effects [288–291]. Statins have been shown to inhibit GGPP biosynthesis, and signal transduction via the Ras/ERK and Ras/Akt pathways, thereby suppressing the expression of basic fibroblast growth factor (bFGF), hepatocyte growth factor (HGF) and transforming growth factor-β (TGF-β) in LM8 cells. These results suggest that statins are potentially useful as anti-angiogenic agents for the treatment of osteosarcoma [121].

In the acidic niche, statins inhibited the proliferation of the human synovial sarcoma cell line SW982 by reducing the production of GGPP [292]. Many studies have shown that statins can influence and regulate the central and the peripheral nervous systems [293–295]. However, there is no conclusive evidence to show that statins exert anti-cancer effects by targeting the innervated niche, and further studies are required to clarify this issue.

Thus, a large amount of evidence shows that statins can exert anti-tumor properties by targeting TME, and repurposing statins to target the TME has shown considerable advantages. These findings indicate that statins can target multiple microenvironments and highlight the great potential for their use in combination with approaches such as immunotherapy, chemotherapy, and targeted therapy.

## Maximizing efficacy and addressing shortcomings of conventional cancer therapy

In recent years, many clinical and preclinical studies have shown the potential of statins to improve the efficacy of various cancer treatments when delivered before or in combination with other therapeutic interventions. Consequently, statins are increasingly considered as potential adjuvant agents in the treatment of cancer. In this regard, statins have unique advantages in that they are safe, well-tolerated and inexpensive, indicating that repurposing these agents may yield a cost-effective, low-toxicity adjuvant therapy for cancer patients. However, in the era of precision medicine, further investigation into drug combination strategies will remain an important area of research [296]. Here, we discuss not only the effects of various statins combination strategies for cancer therapy, but also their unique benefits in terms of addressing the shortcomings of conventional cancer therapy.

In preclinical studies, statins have shown great promise in combined therapies and may act synergistically with some types of widely used forms of chemotherapy. For example, using in vitro and in vivo metabolism tracking, McGregor et al. recently showed that pancreatic ductal adenocarcinoma (PDAC) tumors rely on CoQ synthesized by the mevalonate pathway, and that statins induce cellular oxidative stress via this pathway. Furthermore, simvastatin combined with mitogen-activated protein kinase (MEK) inhibitors was found to significantly enhance pancreatic tumor apoptosis in mice [297]. Taccioli et al. identified that dasatinib and statins are an effective combined strategy for inhibiting YAP/TAZ in cancer cells by interrogating the Mutations and Drugs Portal (MDP) [298]. Iannelli et al. demonstrated the ability of the valproic acid/simvastatin combination to sensitize metastatic castration-resistant prostate cancer cells to docetaxel and to revert docetaxel-resistance through mevalonate pathway/YAP axis modulation using both in vitro and in vivo models [299]. Adriamycin-based chemotherapy is the first-line treatment for osteosarcoma, but most patients will experience tumor recurrence and metastasis. Adriamycin treatment has been shown to induce a stem-like phenotype and promote metastatic potential in osteosarcoma cells by upregulating the Yamanaka factor KLF4. In addition, statins significantly reversed adriamycin-induced cancer stem cell properties and metastasis by downregulating KLF4. Simvastatin also significantly inhibited adriamycin-enhanced tumorigenesis of KHOS/NP cells in vivo. These data indicate that the combined use of statins should be considered for selective inhibition of KLF4 in the development of osteosarcoma therapeutics [300]. Similarly, as the main therapeutic strategy for colorectal cancer, 5-FU-based treatment options have some shortcomings, including the risk of chemotherapy resistance. Recent evidence suggests that simvastatin may enhance the sensitivity of C26 mouse colon cancer cells to 5-FU treatment [301]. Pereira et al. found that statins temporarily modulated the epidermal growth factor receptor (EGFR) and prostate specific membrane antigen (PSMA) on the surface of tumor cells, which enhanced the tumor-binding avidity of the monoclonal antibodies panitumumab, cetuximab and huJ591, thereby synergizing with the antitumor effects of these agents [302]. It has also been reported that lovastatin enhanced the sensitivity of gallbladder cancer to cisplatin [303].

Similarly, in clinical studies, statins have been shown to be effective in adjuvant cancer treatment. Recent clinical studies have shown that high-intensity statins are associated with improved clinical activity of PD-1 inhibitors, and the combined application can improve the prognosis of patients with malignant pleural mesothelioma and advanced non-small cell lung cancer [69]. A recent cohort study from Taiwan has shown that statins are associated with increased survival in lung cancer patients treated with epidermal growth factor receptor-tyrosine kinase inhibitors (EGFR-TKIs) and exert synergistic anti-cancer effects [304]. Similarly, another cohort study of 1,835 patients with gastric cancer from Taiwan, suggested that the statin use improves the overall survival of patients with gastric cancer after surgery and adjuvant chemotherapy [60]. A recent multicenter observational retrospective study showed that the use of statins was independently associated with an increased objective response rate (ORR) of cancer patients treated with PD-1/PD-L1 inhibitors [305]. Interestingly, both metformin and statins significantly reduced prostate cancer tumor invasiveness, and this effect was enhanced (in vitro and vivo) when used in combination [306]. Recently, Longo et al. also found that phosphodiesterase (PDE) inhibitors can enhance statin-induced apoptosis, which may pave the way for the combination of PDE inhibitors and statins in the treatment of hematological malignancies [307].Therefore, a large body of preclinical and clinical evidence shows that statins play a synergistic anti-tumor role when administered in combination with conventional cancer therapy. This evidence may provide references for the inclusion of statins in the future cancer therapy.

Interestingly, statins can not only maximize the efficacy of conventional cancer therapy, but also address the shortcomings of conventional cancer therapy. Although great progress has been made in the field of cancer therapy, chemotherapy and radiotherapy remain the mainstay of cancer therapeutic modalities are extensively used in combination with surgery. While radiotherapy and chemotherapy can improve the survival of cancer patients, these treatments are also associated with serious shortcomings that influence the quality of life of cancer patients. Despite great efforts in the development of new cancer treatment strategies, limited attention has been paid to addressing the shortcomings of cancer treatment. Therefore, in addition to improving survival rates, clinical interventions are urgently needed to minimize the shortcomings and side-effects induced by radiotherapy and chemotherapy and improve the quality of life of cancer patients. In recent years, statins have attracted widespread attention due not only to their anti-tumor properties, but also their potential to address the shortcomings and side-effects of radiotherapy and chemotherapy.

Cisplatin is one of the most widely used anticancer drugs in the treatment of a variety of malignant tumors. It is also the most ototoxic drug in clinical use, causing permanent hearing loss in approximately 50% of treated patients [308–312]. A recent study showed that atorvastatin significantly reduced the incidence and severity of cisplatin-induced hearing loss [313]. In a propensity score-matched cohort study, statin-exposed women had a lower risk of heart failure (HF)-related hospital presentations after anthracycline chemotherapy for early breast cancer, with non-significant trends toward lower risk following treatment with trastuzumab. These findings support the ability of statins to prevent the cardiotoxicity of chemotherapy [314]. In addition, a fraction of patients undergoing androgen deprivation therapy for advanced prostate cancer will develop recurrent castrate-resistant prostate cancer in bone. But recently, Pan et al. found that statins can reduce castration-induced bone marrow adiposity and prostate cancer progression in bone [315]. The safety and toxicity profiles of statins also represent advantages for their use as adjuvant therapy in addressing the shortcomings and side-effects of cancer treatments. Statins used alone and in combined regimens have shown unparalleled advantages as anti-cancer agents and addressing the shortcomings and side-effects of conventional cancer treatments. However, the molecular mechanisms by which statins address these shortcomings and side-effects require clarification to provide a basis for the rational combination of statins and anticancer drugs to optimize cancer therapy.

## Conclusions and future perspectives

Statins belong to a class of lipid-lowering drugs that were originally developed to treat cardiovascular disease. In recent decades, statins have been found to have non-lipid-related effects and are widely recognized as pleiotropic drugs. The anti-tumor properties of statins have received the most attention and have been confirmed in numerous preclinical studies. Here, we have summarized the latest information relating to the anti-tumor mechanisms of statins. The mevalonate pathway was the first anti-tumor mechanism of statins to be discovered and is also the most comprehensively characterized. Statins modulate cell proliferation, differentiation, and apoptosis by depletion of mevalonate pathway intermediates, which interferes with the post-translational modification and activation of small GTPases and their downstream signaling. In addition, the mevalonate pathway is required for the de novo biosynthesis of cholesterol and several studies have shown that cellular cholesterol levels are significantly associated with cancer. Therefore, we have described the role of the mevalonate pathway in anti-tumor mechanism of statins from the perspectives of the cholesterol-mediated pathway and the non-cholesterol-mediated pathways. Moreover, we have summarized the latest discoveries that further clarify the anti-tumor mechanisms of statins, including autophagy, ferroptosis, targeting the tumor microenvironment, and pyroptosis. The improved understanding of these mechanisms will help elucidate the anti-tumor properties of statins and guide clinical trials of statins, thus facilitating the identification of novel cancer therapies.

In recent years, a large number of clinical and epidemiological studies on statins have been conducted, yielding both positive and negative results. Therefore, more well-designed clinical trials are required to validate the anti-cancer effects of statins. However, the value of statins as therapeutic agents against cancer in humans remains an area of active research. Although the clinical evidence that supports the use of statins as a monotherapy for cancer is not convincing, several preclinical and clinical studies indicate that statins potentiate the effects of currently used cancer therapies when administered in combination. Furthermore, statins have been shown to address the shortcomings and side-effects caused by anticancer agents. Nevertheless, difficulties in statin administration at high doses remain unsolved, so satins are currently unlikely to be prescribed as a monotherapy. Therefore, we advocate the use of statins as an adjuvant therapy for cancer, which may be a more promising strategy than statin monotherapy. In addition, the large number of preclinical studies that have provided evidence of the anti-cancer properties of statins, as well as their safety and lack of toxicity, indicate that statins are ready to be investigated in well-designed prospective clinical trials, with lipophilic statins likely leading the charge.

In conclusion, statins are a potential adjuvant to cancer therapy, and more well-designed clinical trials are required to translate this potential to benefit patients.

## Data Availability

Not applicable.

## Abbreviations

ABC: ATP-binding cassette
ABCA1: ATP-binding cassette subfamily A member 1
ABCG1: ABC subfamily G member 1
ACAT1: Acyl-CoA:cholesteryl acyltransferase 1
AKT: Protein kinase B
AML: Acute myeloid leukemia
AMPK: AMP-activated protein kinase
ApoA-I: Apolipoprotein A-I
ATG: Autophagy-related gene
bFGF: Basic fibroblast growth factor
BH4: Tetrahydrobiopterin
Bim: Bcl-2 interacting mediator of cell death
BMP: Bone morphogenetic protein
CEs: Cholesteryl esters
CI: Confidence interval
CoQ10: Ubiquinone
CRC: Colorectal cancer
CSCs: Cancer stem cells
DDS: Disease-specific survival
DNMT: DNA methyltransferase
EAC: Esophageal adenocarcinoma
ECC: Epirubicin, cisplatin and capecitabine
ECM: Extracellular matrix
EGFR: Epidermal growth factor receptor
EGFR-TKIs: Epidermal growth factor receptor-tyrosine kinase inhibitors
EMT: Epithelial-to-mesenchymal transition
ER: Endoplasmic reticulum
ERK: Extracellular regulated protein kinases
FAC: Fluorouracil, adriamycin and cyclophosphamide
FDPS: Farnesyl diphosphate synthase
FPP: Farnesyl pyrophosphate
FSP1: Ferroptosis suppressor protein 1
FTase: Farnesyltransferase
GBC: Gallbladder cancer
GBM: Glioblastoma
GC: Gastric cancer
GCH1: GTP cyclohydrolase 1
GGPP: Geranylgeranyl pyrophosphate
GGPPS: Geranylgeranyl pyrophosphate synthase
GGTase: Geranyltransferases
GLI1: Glioma-associated oncogene homolog 1
GPCR: G protein-coupled receptor
GPX4: Glutathione peroxidase 4
GSH: Glutathione
GTPases: Guanosine-triphosphate hydrolase
HCC: Hepatocellular carcinoma
HDLs: High-density lipids proteins
HF: Heart failure
HGF: Hepatocyte growth factor
HMG-CoA: 3-Hydroxy-3-methyl-glutaryl-CoA
HMGCR: 3-Hydroxy-3-methyl-glutaryl-CoA reductase
HNSCC: Head and neck squamous cell carcinoma
HR: Hazard ratio
IBC: Inflammatory breast cancer
IL: Interleukin
IPP: Isopentenyl pyrophosphate
LC3II: Light chain 3-phosphatidylethanolamine conjugate
LDL: Low-density lipoprotein
LDLR: Low-density lipoprotein receptor
LDs: Lipid droplets
LKB1: Liver kinase B1
lncRNA: Long non-coding RNA
LPS: Lipopolysaccharide
LXRs: Liver X receptors
MA: Mevalonate
MCT1: Monocarboxylate transporter 1
MDP: Mutations and Drugs Portal
MEK: Mitogen-activated protein kinase
MEV: Mevalonate
MM: Multiple myeloma
mTORC1: Mechanistic target of rapamycin complex 1
NHL: Non-Hodgkin lymphoma
NLRP3: Nod-like receptor protein 3
NR: Not reported
NSCLC: Non-small cell lung cancer
ORR: Objective response rate
OS: Overall survival
PARP: Poly(ADP-ribose) polymerase
PBR: Peripheral benzodiazepine receptor
PCa: Prostate cancer
PCD: Programmed cell death
PCSK9: Proprotein-convertase-subtilisin-kexin type-9
PDAC: Pancreatic ductal adenocarcinoma
PDE: Phosphodiesterase
PD-1: Programmed death receptor-1
PFS: Progression-free survival
PI3K: Phosphatidylinositol-3-kinase
PSMA: Prostate specific membrane antigen
PTEN: Phosphatase and tensin homolog
PTTG1: Pituitary tumor-transforming gene 1
PUFA: Polyunsaturated fatty acid
p38MAPK: P38 mitogen-activated protein kinase
RCC: Renal cell carcinoma
ROCK: Rho-associated protein kinase
ROS: Reactive oxygen species
RR: Relative risk
SCAPs: SREBP cleavage activating proteins
SHH: Sonic Hedgehog
SMO: Smoothened receptor
SRE: Sterol regulatory elements
SREBPs: Sterol regulatory element binding proteins
TAE: Transcatheter arterial embolization
TAM: Tumor-associated macrophages
TGF-β: Transforming growth factor-β
TME: Tumor microenvironment
TMZ: Temozolomide
TRAIL: TNF-related apoptosis inducing ligand
VEGF: Vascular endothelial growth factor
YAP: Yes-associated protein
5-FU: 5-Fluorouracil
Δψm: Mitochondrial membrane potential
